# Transactivation of Met signaling by oncogenic Gnaq drives the evolution of melanoma in Hgf-Cdk4 mice

**DOI:** 10.1038/s41417-024-00744-0

**Published:** 2024-02-15

**Authors:** Miriam Mengoni, Andreas Dominik Braun, Sahithi Seedarala, Susanne Bonifatius, Evi Kostenis, Denny Schanze, Martin Zenker, Thomas Tüting, Evelyn Gaffal

**Affiliations:** 1 Laboratory for Experimental Dermatology, Department of Dermatology, University Hospital Magdeburg, 39120 Magdeburg, Germany; 2 Molecular, Cellular and Pharmacobiology Section, Institute for Pharmaceutical Biology, University of Bonn, Nussallee 6, 53115 Bonn, Germany; 3 Institute of Human Genetics, University Hospital Magdeburg, 39120 Magdeburg, Germany

**Keywords:** Cancer genetics, Melanoma, Cancer models

## Abstract

Recent pan-cancer genomic analyses have identified numerous oncogenic driver mutations that occur in a cell-type and tissue-specific distribution. For example, oncogenic mutations in *Braf* and *Nras* genes arise predominantly in melanocytic neoplasms of the epidermis, while oncogenic mutations in *Gnaq/11* genes arise mostly in melanocytic lesions of the dermis or the uvea. The mechanisms promoting cell-type and tissue-specific oncogenic events currently remain poorly understood. Here, we report that *Gnaq/11* hotspot mutations occur as early oncogenic drivers during the evolution of primary melanomas in Hgf-Cdk4 mice. Additional single base substitutions in the *Trp53* gene and structural chromosomal aberrations favoring amplifications of the chromosomal region containing the Met receptor gene accumulate during serial tumor transplantation and in cell lines established in vitro. Mechanistically, we found that the Gnaq^Q209L^ mutation transactivates the Met receptor. Overexpression of oncogenic Gnaq^Q209L^ in the immortalized melanocyte cell line promoted in vivo growth that was enhanced by transgenic Hgf expression in the tumor microenvironment. This cross-signaling mechanism explains the selection of oncogenic *Gnaq/11* in primary Hgf-Cdk4 melanomas and provides an example of how oncogenic driver mutations, intracellular signaling cascades, and microenvironmental cues cooperate to drive cancer development in a tissue-specific fashion.

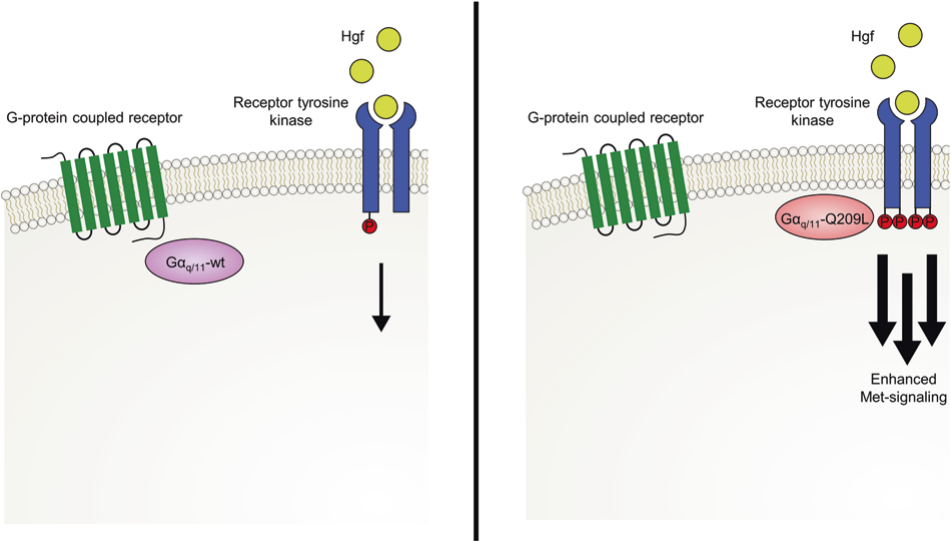

## Introduction

In the last decade, large sequencing studies have generated in-depth insights into the genomic alterations of cancer cells [1]. Interestingly, these analyses revealed a non-random distribution of oncogenic driver mutations in cancer cells arising in different tissues and cell types. For example, nearly one-half of melanomas arising in the epidermis harbor oncogenic mutations in the *BRAF* gene [2, 3], while almost half of the melanomas arising in the uvea harbor oncogenic mutations in the *GNAQ/GNA11* genes [4]. The mechanisms that drive the tissue-specific selection of oncogenic driver mutations in melanoma or other cancer types are poorly understood.

In our previous work, we identified an oncogenic driver mutation in the *Gna11* gene of the HCmel12 mouse melanoma cell line [5]. This cell line was derived from a carcinogen-induced primary melanoma that developed in the skin of an Hgf-Cdk4 mouse [6]. In this genetically engineered melanoma model, transgenic, metallothionein promoter-driven overexpression of the hepatocyte growth factor (Hgf) that deregulates receptor tyrosine kinase signaling is combined with an oncogenic CDK4^R24C^ germline mutation that impairs cell cycle control. These mice harbor a “chocolate point phenotype” due to an accumulation of melanocytes in the skin [7]. The genetic alterations that are present in all cells of these mice favor the spontaneous development of cutaneous melanomas [8]. Exposure to sun-burning doses of Ultraviolet B light (UVB) irradiation or to the chemical carcinogen 7,12-Dimethylbenz[a]anthracene (DMBA) accelerates melanoma genesis [7, 9], and repetitive application of inflammatory stimuli to the skin promotes metastatic spread [6, 10].

Here, we report the evolutionary trajectory of genomic alterations that drive primary cutaneous and serially transplanted melanomas of Hgf-Cdk4 mice. The selection of the oncogenic hotspot mutation in the *Gnaq/11* gene that promotes Hgf-Met cross-signaling in Hgf-transgenic mice provides an example of how oncogenic driver mutations, intracellular signaling cascades, and microenvironmental cues can cooperate to drive cancer development in a tissue-specific fashion.

## Materials and methods

### Cell culture

HCmel cell lines were derived from Hgf-Cdk4^R24C^ mice as previously described [6, 11]. BCmel4 was derived from the Braf^V600E^-Cdk4^R24C^ model as previously described [12]. Melan-a cells were kindly provided by D. Bennett (London, United Kingdom). All HCmel cell lines, BCmel4 and Melan-a cells were cultured in RPMI 1640 medium (Life Technologies, Carlsbad, CA) supplemented with 10% fetal bovine serum (Biochrome, Berlin, Germany), 2 mM L-glutamine, 10 mM non-essential amino acids, 1 mM 4-(2-hydroxyethyl)-1-piperazineethanesulfonic acid (HEPES), 100 U/mL penicillin and 100 μg/mL streptomycin (all from Life Technologies, Carlsbad, CA) and 20 μM 2-mercaptoethanol (Sigma, St. Louis, MO) in a humidified incubator with 5% CO2 at 37 °C. For culturing of Melan-A cells additionally, 200 nM Phorbol 12-myristate 13-acetate (PMA) (Sigma) was supplemented into the medium. All cells were cultured at 37 °C and 5% CO_2_ in a humid environment.

For pharmacologic manipulation, cells were treated with recombinant mouse HGF protein (R&D, Minneapolis, MI), the MET inhibitor capmatinib (Cayman Chemical, Ann Arbor, MI), and the Gnaq-inhibitor FR900359 (FR) (kindly provided by E. Kostenis, Bonn, Germany) as described in the figure legends. Vehicle controls were performed with DMSO (Sigma) in a final concentration of 0.1%. For the retrovirus production, HEK293T (RRID: CVCL_0063) cells, obtained from the American Type Culture Collection (ATCC), were maintained in DMEM medium (Life Technologies) containing the same supplements. Cells were freshly thawed every 2 months. All cell lines used in our study were routinely tested for mycoplasma contamination via polymerase chain reaction (PCR) monthly. All experiments were performed with mycoplasma-free cells.

### Cloning of pRP-mGnaq^WT^/mGnaq^Q209L^-T2A-mcherry plasmids and retroviral gene transfer

For generation of the retroviral overexpression constructs of Gnaq^WT^ and Gnaq^Q209L^, the Gnaq cDNA was amplified from cDNA from HCmel3 cells, which are heterozygous for Gnaq^209L^ using primers C1 and C2 (see Table S1). A fragment encoding T2A-mCherry was amplified via PCR using the plasmid pCAS9-mCherry-Frame +0 (Addgene #66939) containing mCherry as the template using the primers C3 and C4 (containing the T2A-sequence, see Table S1). The retroviral backbone pRP (Addgene #41841) was linearized using the restriction enzyme XhoI. The correct size of all fragments was verified via agarose gel electrophoresis with subsequent isolation of DNA using the NucleoSpin Gel and PCR clean-up kit (Macherey Nagel, Düren, Germany) according to manufacturer’s instructions. Subsequently, all fragments were assembled in a sequence-independent cloning reaction using the Gibson assembly master mix (New England Biolabs, Ipswich, MA) according to manufacturer’s recommendations.

HEK293T cells were transfected with the retroviral helper plasmids (gag-pol [Addgene plasmid #14888] and pCMV VSV-G [Addgene plasmid #14887], kindly provided by E. Latz, Bonn, Germany) and pRP-TagGFP2 by calcium phosphate transfection according to standard protocols. Retrovirus-containing supernatant was filtered using a 0.45 μM pore size syringe filter and added to target cells. Selection with 10 μg/mL puromycin (AppliChem, Darmstadt, Germany) was started 48 hours after transduction for 5 days. Successful transduction was confirmed by red fluorescence using a Zeiss AxioVert A1 microscope (Zeiss, Oberkochen, Germany).

### Crystal violet assay

Cells were seeded at a density of 2 × 10^4^ cells/well in biological triplicates in a 48-well plate and treated as indicated the next day. Subsequently, cells were incubated as described above. After this time, media was removed, and wells were washed with 1× phosphate-buffered saline (PBS) to remove dead cells. All wells were stained with 150 µL of 0.5% crystal violet in aqueous solution for 10 min, repetitively washed three times with water, and dried at room temperature for 48 hours. Images were acquired with an EOS 750D camera (Canon, Tokyo, Japan).

### Immunoblot analysis

Whole-cell lysates were extracted from cultured cells using the M-PER mammalian protein reagent supplemented with protease and phosphatase inhibitors (all Thermo Fisher Scientific, Waltham, MA) according to the manufacturer’s protocol. Protein concentrations were quantified with a Pierce BCA Protein Assay Kit (Thermo Fisher Scientific) at 562 nm in a microplate reader (Tecan Group, Männedorf, Switzerland). Samples were prepared containing 2 μg protein, Roti-Load (Carl Roth, Karlsruhe, Germany), and nuclease-free water and denatured at 95 °C prior to loading. In the next step, samples were separated via 10% SDS-PAGE and transferred to a 0.45 μM polyvinylidene difluoride membrane (GE Healthcare, Boston, MA) by wet blotting (BioRad, Hercules, CA). After blocking with 5% milk (Carl Roth), blots were immunostained at 4 °C overnight. Bound antibodies were detected with horseradish peroxidase (HRP)-conjugated secondary antibodies and the SignalFire ECL Reagent (Cell Signaling, Danvers, MA) according to the manufacturer’s instructions. Chemiluminescence was visualized using an Octoplus QPLEX-Imager (NH DyeAGNOSTICS, Halle, Germany). Used antibodies were as follows: rabbit anti-mouse phospho-Met antibody (Cell Signaling, #3077), rabbit anti-mouse phospho-c-Met polyclonal antibody (Thermo Fisher Scientific, #44-888 G), mouse anti-mouse Met monoclonal antibody (Cell Signaling, #3127), mouse β-Actin monoclonal antibody (Santa Cruz, Dallas, TX, sc-47 778) and goat anti-rabbit IgG HRP-linked antibody (Cell Signaling, #7074 S).

### Next-generation sequencing and data analysis

DNA was isolated from mouse tumor tissue or cultured cells using the NucleoSpin Tissue kit according to manufacturer’s instructions (Macherey Nagel). Target DNA regions for *Gnaq/11* Exon 4 and 5, as well as Trp53 Exon 4–7 were amplified and supplemented with unique dual sequencing indices and adapters in a two-step PCR (Primer sequences see Table S1). Samples were multiplexed and sequenced on an Illumina MiSeq in paired-end mode, sequencing length of 150 bp and minimum depth of 100×. Samples were demultiplexed based on their unique sequencing indices, and quality control was performed separately for each sample using fastqc. Samples were aligned to the GRCm39 reference genome using bwa in mem-mode. Variant calling was performed with mutect2 without positional downsampling.

### Analysis of published datasets

Met copy number and transcript data were obtained from the DepMap portal from the 2023Q2 release [13]. Whole-genome sequencing data from HCmel12 cells as published in [14], available under the accession SRP247646 were downloaded from the Sequence Read Archive as fastq files. Raw reads were aligned to the mm9 reference genome using BWA (version 0.7.17) using the BWA-MEM algorithm with default parameters. Duplicate marking was performed with GATK (version 4.4.0.0) using the command MarkDuplicatesSpark with default parameters. For variant calling, cnvkit (version 0.9.10) was used using the batch command using a “flat” reference of neutral copy number by providing the “n” flag without specifying a normal sample. Discrete copy number segmentation was performed with cnvkit segment using the cbs method and the “drop-low-coverage” flag. The absolute integer copy number was assigned using cnvkit call using the “clonal” method and assuming a purity of 95%.

### Comparative genomic hybridization (CGH)

DNA was fluorescence-labeled using genomic DNA universal linkage system labeling kits (Agilent Technologies, Böblingen, Germany) and was hybridized using an Agilent mouse genome CGH 2x105k microarray (Agilent Technologies) according to the manufacturer’s instructions. The resolution of the CGH arrays was 2×105,000 oligonucleotides distributed genome-wide. The arrays were scanned using a DNA microarray scanner (Agilent Technologies), and the images were analyzed using Feature Extraction, version 10.5.1.1, and DNA Analytics software, version 4.0.85 (Agilent Technologies) based on the mouse genome build mm8.

### Animal experiments

Hgf-Cdk4- and Cdk4 mice were taken from their own breeding [7, 15, 16]. Age- and sex-matched cohorts of mice were randomly allocated to the different experimental groups at the start of each experiment. Experiments were performed with 6-8-week-old mice. Induction of primary melanoma was performed with a single dose of 100 nmol DMBA dissolved in acetone applied to the shaved back skin as previously described [11]. UV irradiation was performed twice weekly with 4.5 kJ/m^2^ dose applied to the shaved back using a UV 302 L system (Waldmann, Villingen-Schwenningen, Germany) equipped with eight 100 W UV21 lamps (Phillips, Amsterdam, Netherlands) [6]. For tumor cell transplantation, cohorts of syngeneic Hgf-Cdk4- and Cdk4 mice were injected intracutaneously with 2 × 10^5^ cells resuspended in 100 μL PBS (Life Technologies) into the right flank. Tumor growth was monitored by inspection and palpation. Tumor size was measured at least twice times weekly with a vernier caliper and recorded as the mean diameter of two perpendicular measurements. Mice were sacrificed when tumors exceeded 20 mm in diameter or when signs of illness were observed. All experiments were performed in groups of five or more mice and repeated independently three times. The sample size for animal experiments was determined based on our own previous experience of tumor engraftment and growth rates. No animals were excluded from the analysis. Tissue samples for histological analysis were fixed in formalin free Zinc-fixative (BD Bioscience) and subsequently prepared for sectioning and staining using routine histological techniques. Images were obtained using an AxioVert A1 Microscope (Zeiss). All experiments were performed in compliance with federal and international guidelines for animal experiments and with the approval of the responsible authorities (Landesverwaltungsamt Saxony-Anhalt, Germany, approval number: 42502-2-1556 UniMD).

### Statistical analysis

Survival analyses were performed using Kaplan-Meier estimators with pairwise log-rank-tests between experimental cohorts. Tumor growth curves for individual tumors are additionally shown to demonstrate a similar variance between experimental cohorts. All bioinformatical and statistical analyses were performed using Python 3.9 and the packages biopython, pandas, scikit-allel, and lifelines.

## Results

### Hgf-Cdk4 melanoma cells can be readily propagated as serial tumor transplants in vivo but only rarely grow in tissue culture in vitro

**Fig. 1:**
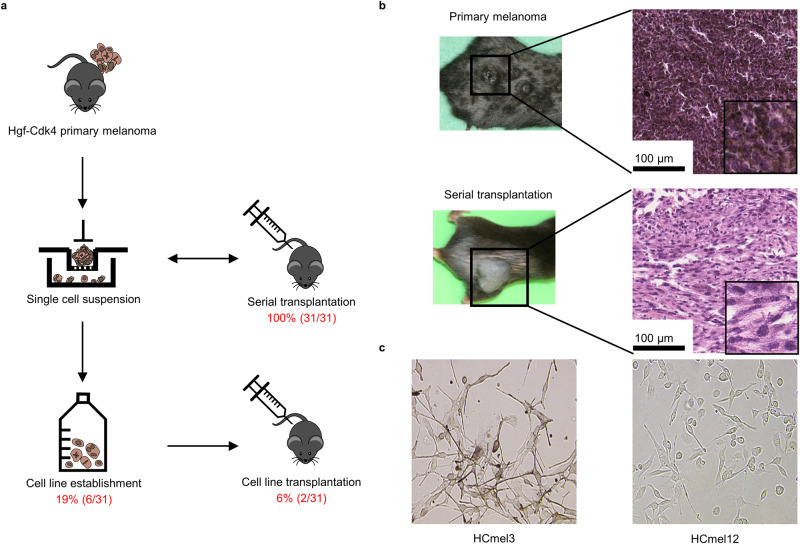
Hgf-Cdk4 melanoma cells can be readily grown as serial tumor transplants in vivo but only rarely establish as transplantable cell lines in vitro. **a** Experimental protocol for the establishment of serial tumor transplants and cell lines. **b** Representative macroscopic and microscopic images of primary and serially transplanted Hgf-Cdk4 melanomas. The magnification of micrographs is shown in each panel. **c** Representative phase contrast images of cultured HCmel3 and HCmel12 cells.

In previous work, we established the genetically engineered Hgf-Cdk4 mouse melanoma model, in which mice spontaneously develop melanocytic nevi and metastatic melanoma in a stepwise progression [8, 9]. While Hgf-Cdk4 melanoma cell suspensions could be readily propagated as serial tumor transplants in vivo [6], they only very rarely established in tissue culture in vitro (Fig. 1a). Histological examination of serial transplants revealed a distinct morphology with an increase in spindle-shaped cell phenotypes and a decrease in melanin pigment over time when compared to the predominance of epitheloid, highly pigmented cell phenotypes in primary melanomas (Fig. 1b). The rare cell cultures that could be established from single tumor cell suspensions recapitulated the phenotypic spectrum of primary and transplanted melanomas (Fig. 1c).

### Melanoma cells harboring oncogenic Gnaq/11 mutations are selected early during tumor evolution in Hgf-Cdk4 mice

**Fig. 2:**
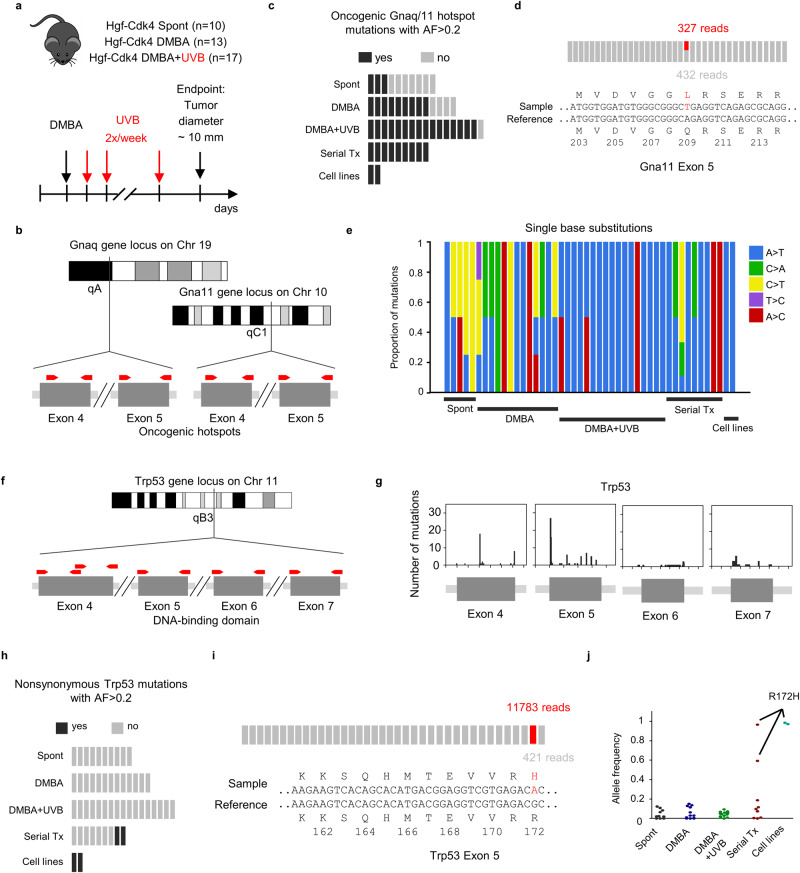
Gnaq/11 hotspot mutations occur as an early oncogenic driver in primary cutaneous melanomas of Hgf-Cdk4 mice, whereas non-synonymous Trp53 mutations accumulate later during serial tumor transplantation and cell lines established in vitro. **a** Experimental protocol of the primary melanoma models. **b** Sequencing strategy: depicted are the genetic loci of Gnaq/11 genes. Red arrows denote the location of the primer pairs used for the amplification prior to sequencing. **c** Occurrence of Gnaq/11 hotspot mutations with an allele frequency >0.2 over all sequenced specimen types with each bar representing a single sample. **d** Example of alignment with the heterozygous Gna11 Q209L mutation. Reference reads are labeled in gray, mutated reads are labeled in red. **e** Proportion of the subtypes of single base substitutions for all sequenced samples ordered by type of specimen. **f** Sequencing strategy: depicted are the genetic loci of the Trp53 gene. Red arrows denote the location of the primer pairs used for the amplification prior to sequencing. **g** Histogram showing the distribution of non-synonymous mutations over exons 4–7 of Trp53 from all samples combined. **h** Occurrence of non-synonymous Trp53 mutations with an allele frequency >0.2 over all sequenced specimen types, with each bar representing a single sample. Each dot indicates a single variant. **i** Example of alignment with the homozygous Trp53 R172H mutation. Reference reads are labeled in gray, mutated reads are labeled in red. **j** Stripplot indicating the allele frequency of non-synonymous Trp53 mutations. Highlighted are all R172H mutations.

Hotspot mutations in *Braf*, *Nras*, and *Gnaq/11* genes represent frequent oncogenic driver events early in the evolution of melanomas. Additional oncogenic events are selected during disease progression. In previous work, we identified the oncogenic Q209L mutation in the Hgf-Cdk4 melanoma cell line HCmel12 [5], raising the question of whether *Gnaq* mutations are a frequent early oncogenic driver event during melanoma evolution in the Hgf-Cdk4 melanoma model. To address this hypothesis, we isolated genomic DNA from archived cryopreserved and paraffin-embedded tissue specimen of primary cutaneous melanomas obtained from untreated, DMBA-treated, and DMBA + UVB-treated Hgf-Cdk4 mice, from serially transplanted tumors, and from the HCmel12 and HCmel3 melanoma cell lines (Fig. 2a). Genomic regions covering the oncogenic hotspots of *Gna11* and *Gnaq* were amplified via PCR and sequenced using next-generation sequencing (Fig. 2b). As a cutoff for putative heterozygous mutations, a frequency of 20% was chosen to account for potential whole-genome doubling events. Many primary melanomas and all serial transplants and cell lines harbored high-frequency *Gnaq/Gna11* Q209 hotspot mutations (Fig. 2c, d, Supplementary Table 1), Mutations in the R183 position that are also occasionally observed in human melanomas were not observed. The composition of single base substitutions shows an accumulation of A > T transversions (Fig. 2e), representing the underlying substitution responsible for the frequently observed Gnaq/11 Q209L mutation. Additionally, A > T transversions have been described as a typical signature for DMBA-induced mutations [17]. These results indicate a positive selection of oncogenic *Gnaq/11* mutations early during melanoma development in Hgf-Cdk4 mice.

### Non-synonymous Trp53 mutations in Hgf-Cdk4 mouse melanomas accumulate during serial tumor transplantation in syngeneic mice

Experimental evidence in melanoma mouse models has linked *Trp53* mutations to UV damage and melanoma progression [18]. We therefore performed sequencing of the *Trp53* gene exons 4–7, encoding large parts of the DNA binding domain (Fig. 2f). A variety of non-synonymous mutations were detected at low frequency in exons 4 and 5 (Fig. 2g, Supplementary Table 1). In serial transplants and cell lines but not in primary tumors, we observed high-frequency non-synonymous mutations in *Trp53* (Fig. 2h). An increase in the allele frequency of the mutated *Trp53* allele during serial transplantation further highlights the genetic selection of *Trp53* mutations during evolution (Supplementary Fig. 1). The homozygous R172H mutation was found in the HCmel12 cell line and a serially transplanted tumor (Fig. 2i, j, Supplementary Table 1). This mutation represents a hotspot that is also frequently observed in human tumors.

### Structural chromosomal aberrations increase during serial transplantation and favor amplification of the chromosomal region containing the Met receptor gene

**Fig. 3:**
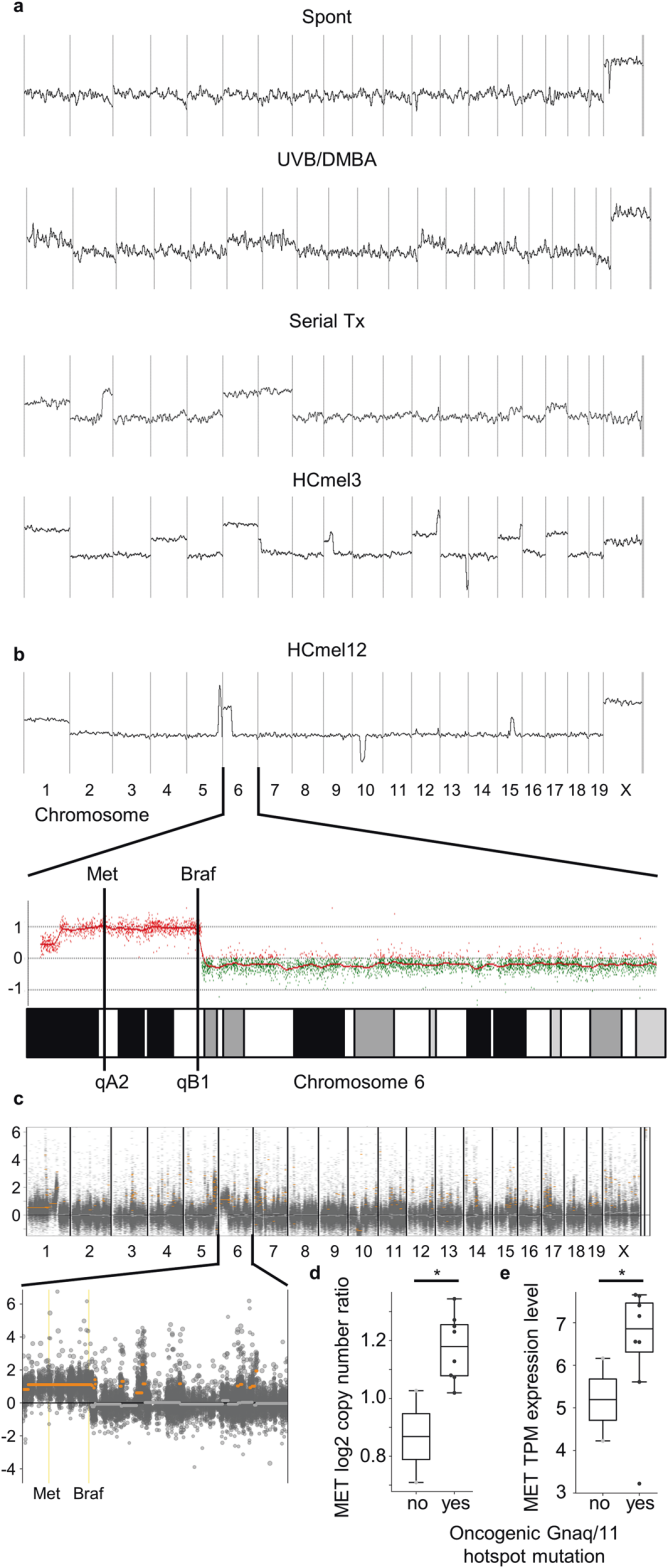
Structural chromosomal aberrations increase during serial transplantation and favor an amplification of the chromosomal region containing the Met receptor gene. **a** Representative comparative genomic hybridization results for Hgf-Cdk4 primary melanomas, serial tumor transplants, and the melanoma cell line HCmel3. Lines indicate the copy number state at the indicated location. **b** Copy number state of the HCmel12 cell line. The bottom part highlights chromosome 6 and the respective genomic amplification containing the Met and Braf gene loci. **c** Copy number state of the HCmel12 cell line as derived from previously published whole exome sequencing (SRA accession SRP247646). The top panel shows overview over all chromosomes, the bottom panel demonstrates the copy number state of the Met and Braf locus. **d** MET copy number state of 10 uveal melanoma cell lines from the DepMap project stratified by the presence of oncogenic Gnaq/11 Q209 mutations. **e** MET expression levels of the uveal melanoma cell lines from **d** stratified by the presence of oncogenic Gnaq/11 Q209 mutations. Values shown are transcripts per million (TPM). (*p < 0.05, *t* test).

Recent advances in large cancer sequencing studies have highlighted the importance of structural aberrations in cancer development. To analyze the occurrence of structural aberrations in the Hgf-Cdk4 melanoma model, we performed comparative genomic hybridization analyses of primary melanomas, serial transplants, and the HCmel3 and HCmel12 cell lines. We observed only very few structural genomic aberrations in primary melanomas that arose spontaneously in untreated Hgf-Cdk4 mice. Primary melanomas from UVB/DMBA-treated Hgf-Cdk4 mice showed initial large chromosomal gains and losses that markedly increased during serial transplantation and in established cell lines (Fig. 3a). The genomic amplification favored a region of chromosome 6 between qA2 and qB1 that contains both the Met and the Braf gene (Fig. 3b). The amplification of the Met and Braf locus in HCmel12 cells was confirmed also in a previously published independent whole exome sequencing dataset [14] (Fig. 3c). Furthermore, we observed a significantly higher copy number of the MET gene in association with enhanced MET transcript levels in cell lines harboring Gnaq/11 Q209 mutations in a dataset of 10 uveal melanoma cell lines from the DepMap project [13] (Fig. 3d, e). This indicates that genomic alterations that increase autochthonous Hgf-Met signaling are selected during the evolution of transplanted melanomas and melanoma cell lines, potentially as a result of genomic instability caused by the acquisition of Trp53 mutations observed concurrently during serial transplantation.

### Oncogenic activation of Gnaq transactivates the Met receptor in melanoma cells

**Fig. 4:**
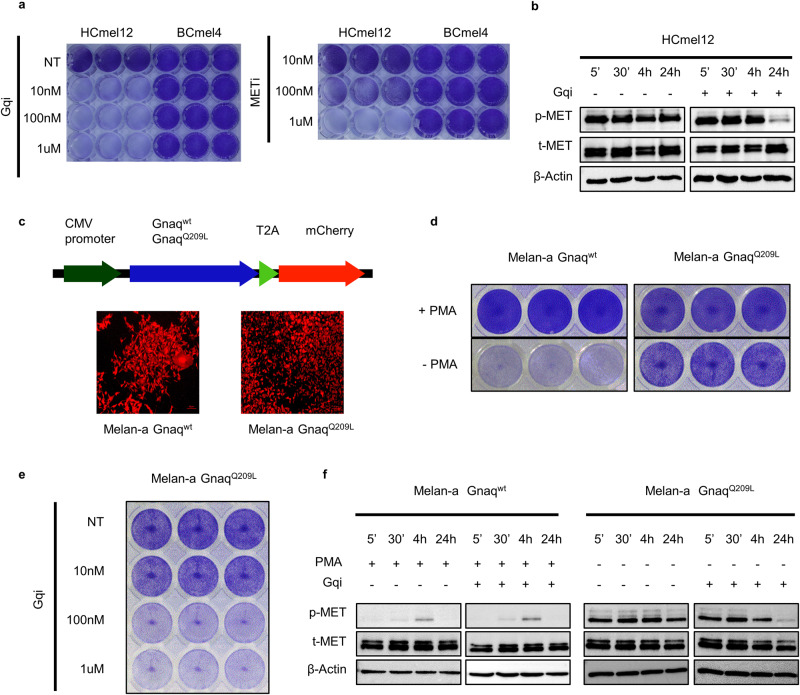
The oncogenic GnaqQ209L mutation transactivates the Met receptor in mouse HCmel12 melanoma cells and in the immortalized melanocyte line Melan-a. **a** Crystal violet assay of HCmel12 cells treated with the Gnaq-Inhibitor FR900359 (Gqi) or the Met-Inhibitor Capmatinib (METi) as indicated for 72 h. **b** Western blot of HCmel12 cells treated with the Gnaq-Inhibitor FR900359 or vehicle for the time indicated. **c** Schematic overview of the retroviral CMV-promoter-driven Gnaq^wt^ and Gnaq^Q209L^ overexpression vectors (top) with fluorescence microscopy images of transduced cells (bottom). **d** Crystal violet assay of Melan-a cells transduced with the Gnaq^wt^ and Gnaq^Q209L^ overexpression vectors treated for 72 h with and without PMA. **e** Crystal violet assay of Melan-a Gnaq^Q209L^ cells treated with the Gnaq-Inhibitior FR900359 for 72 h in the indicated concentrations. **f** Western blot of Melan-a Gnaq^wt^ and Gnaq^Q209^ cells treated with PMA and FR900359 for the indicated times. All experiments were performed in triplicates, data shown are representative images from one experiment.

The frequent occurrence of oncogenic Gnaq Q209 mutations in Hgf-Cdk4 melanomas suggested a mechanistic connection between mutated Gnaq and Hgf-Met signaling. Pharmacologic inhibition of Gnaq with the compound FR900359 and of Met signaling with Capmatinib abrogated growth of HCmel12 melanoma cells, indicating that the activity of both pathways was required for cell proliferation (Fig. 4a). Interestingly, the inhibition of Gnaq abrogated the phosphorylation of the Met receptor in HCmel12 cells after 24 h (Fig. 4b), suggesting a direct activation of Met signaling by mutated Gnaq. To validate the interaction between mutated Gnaq and Met also in another melanocytic model without transgenic Hgf overexpression, we transduced the immortalized melanocyte line Melan-a with retroviral constructs expressing wildtype or mutated Gnaq (Fig. 4c). Melan-a Gnaq^Q209L^ but not Melan-a Gnaq^wt^ cells were able to grow in the absence of the tumor promotor phorbol 12-myristate 13-acetate (PMA, Fig. 4d). Growth of Melan-a Gnaq^Q209L^ cells was sensitive to inhibition of Gnaq signaling (Fig. 4e). Overexpression of Gnaq^Q209^ in Melan-a cells caused constitutive phosphorylation of the Met receptor which was abrogated after Gnaq inhibition (Fig. 4f). We did not observe significant differences in the downstream signaling pathways of Met, namely Akt, Stat3 or Fak in HCmel12 cells after treatment with the Met inhibitor Capmatinib (Supplementary Fig. 2). We furthermore expanded our cytotoxicity assays to include an independent assay of uveal melanoma cell lines. In none of the cell lines tested, we identified significant cytotoxicity of cells exposed to the Met inhibitor capmatinib, regardless of the presence of Gnaq/11 Q209 mutations (Supplementary Fig. 3). In summary, these observations support the notion that oncogenic Gnaq leads to transactivation of the Met receptor in melanocytic cells.

### The oncogenic Gnaq mutation endows incipient melanoma cells with increased responsiveness to microenvironmental Hgf

**Fig. 5:**
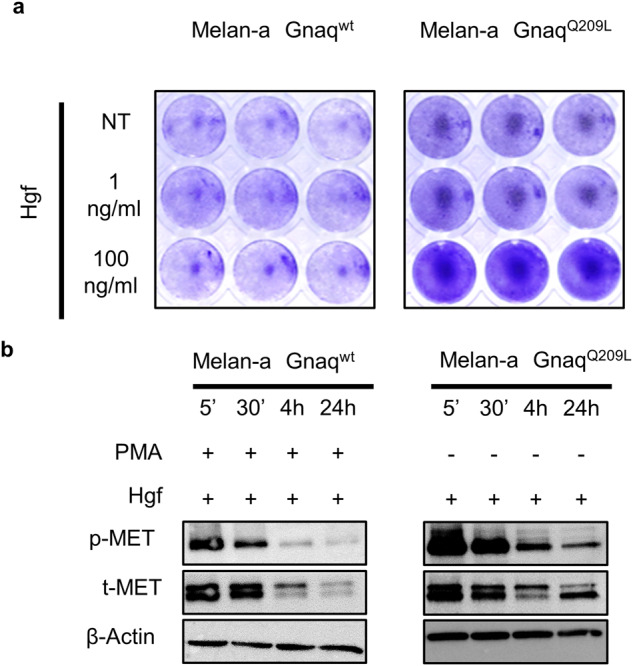
The oncogenic GnaqQ209L mutation sensitizes the immortalized melanocyte line Melan-a for Hgf-Met signaling. **a** Crystal violet assay of Melan-a Gnaq^wt^ and Gnaq^Q209^ cells treated with the indicated concentrations of Hgf for 72 h. **b** Western blot of Melan-a cells expressing Gnaq^wt^ or Gnaq^Q209L^ treated with PMA and/or Hgf for the times indicated. All experiments were performed in triplicates, the data shown are representative images from one experiment.

**Fig. 6:**
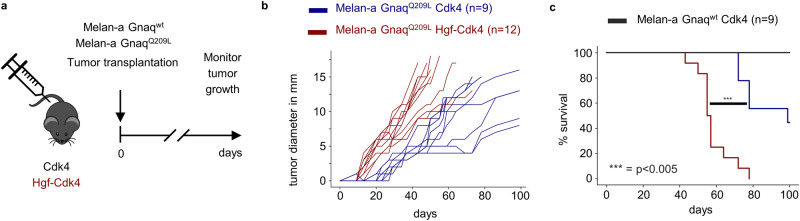
The oncogenic GnaqQ209L mutation enables in vivo growth of Melan-a cells that is accelerated by transgenic expression of Hgf in the tumor microenvironment. **a** Experimental protocol. **b** Individual tumor growth curves of Hgf-Cdk4 and Cdk4 mice transplanted with Melan-a Gnaq^wt^ or Gnaq^Q209L^ cells. **c** Corresponding Kaplan–Meier survival curves (***p < 0.005, logrank test).

After identifying a connection between mutant Gnaq and Met signaling, we hypothesized that oncogenic Gnaq mutations endow incipient melanoma cells with enhanced responsiveness to microenvironmental Hgf. Indeed, treatment of Melan-a Gnaq^Q209L^ cells with Hgf promoted proliferation, whereas it did not affect the growth of Melan-a Gnaq^wt^ cells (Fig. 5a). Treatment of Melan-a Gnaq^Q209L^ cells with Hgf increased the phosphorylation of the Met receptor as compared to Gnaq^wt^ cells (Fig. 5b). Overexpression of Gnaq^Q209L^ but not Gnaq^wt^ in Melan-a cells enabled in vivo growth following transplantation in syngeneic Cdk4 mice (Fig. 6a, b). Importantly, the growth kinetics was increased in Hgf-Cdk4 mice when compared to Cdk4 mice (Fig. 6b), leading to increased tumor penetrance and significantly shorter survival (Fig. 6c). Together, these results indicate that the oncogenic *Gnaq* mutation sensitizes melanocytes to Hgf-Met signaling and provides a growth advantage in a microenvironment rich in Hgf.

## Discussion

In our current work, we identified an evolutionary trajectory of recurrent *Gnaq/11* mutations early during melanoma evolution in Hgf-Cdk4 mice, followed by *Trp53* mutations and structural genomic aberrations. This faithfully recapitulates common steps in cancer evolution with a continuous accumulation of mutations during disease development and progression, driving cancer cell heterogeneity and promoting transformation, growth, and therapy resistance [19]. Conceptually, the dysfunction of p53 serves as a gateway to increasing chromosomal instability and drives the evolution of cancer cells with the emergence of novel subclones [20]. In the Hgf-Cdk4 model, incipient melanomas were selected for amplifications of chromosome 6 in the region harboring the gene encoding the Met receptor. The synergy of the Met-amplification with the model-specific Hgf overexpression suggests that the tumor environment can profoundly modify the landscape of genomic aberrations. Recurrent copy number alterations involving key driver genes have also been described in other cancer entities. As an example, amplifications involving the *KRAS* gene after p53 loss have been observed in pancreatic cancer [21], supporting a broader applicability of the concept of environmental governance of tumor cell-specific copy number alterations. Despite the similarity of our findings with other work performed in human cancer systems, one limitation of our data is its generation using genetically engineered mouse models, model carcinogens as well as artificial transplantation systems. Further work expanding our findings to the human system is required to translate our research into patient benefit.

In our work, we uncover transactivation of Met via oncogenic Gnaq/11, explaining the frequent occurrence and selection of *Gnaq/11* mutations in primary melanomas of Hgf-Cdk4 mice. Interestingly, also melanoma samples from other Hgf-driven mouse models have been demonstrated to harbor *Gnaq/11* mutations, indicating a more general presence of this cross-signaling mechanism [14]. Surprisingly, we did not observe a cytotoxic effect of Met inhibition on a panel of uveal melanoma cell lines regardless of Gnaq/11 mutations present in these cells. As most of these cell lines were derived from primary uveal melanoma specimens, the absence of Hgf in the microenvironment of these cell lines might result in a lack of selection pressure for Met-signaling, explaining their Met-signaling independent growth. We hypothesize that the cross-signaling mechanism identified in our work is most relevant to Hgf-rich environments such as liver metastases.

An activation of the Met-receptor through G-protein coupled receptor (GPCR) signaling has additionally been shown in hepatocellular and pancreatic cancer cells and is hypothesized to be catalyzed through the generation of reactive oxygen species [22]. Other proposed mechanisms of cross-signaling between GPCR and receptor tyrosine kinases (RTK) involve the activation of protein kinases such as c-Src by the G_αq/11_-subunit and subsequent phosphorylation of RTKs [23], or the promotion of matrix metalloproteinases by GPCR-signaling with liberation of ligands such as proHB-EGF [24–26]. Conceptually, these cross-signaling cascades broaden the cellular signaling repertoire in order to fine-tune cell-intrinsic responses to environmental stimuli [26]. Our work identifies these cross-signaling mechanisms as a selective driving force in cancer evolution, underlining the central role of Gnaq signaling in cancer.

The high frequency of Gnaq/11 mutations in blue nevus-like melanoma and uveal melanoma has identified the inhibition of Gnaq signaling as a novel potential therapeutic strategy [27]. Despite this clinical interest, the development of Gnaq/11 inhibitors has proved challenging, in part due to the occurrence of adverse drug reactions [28]. Recently, the inhibition of protein kinase C (PKC) has emerged as a potential target to block oncogenic Gnaq signaling [28–30]. However, the blockade of PKC alone has been limited in its efficacy due to the development of resistance mechanisms, activating PKC-independent signaling cascades downstream of Gnaq [31]. Our data suggest an additional role of Met signaling in Gnaq-driven cancer types, supporting the rationale for a combined PKC and Met inhibition as currently explored in early clinical trials.

Since human blue nevi and derived melanomas situated in the dermis also frequently harbor *Gnaq/11* mutations [4, 32], our results support the idea that cross-signaling between oncogenic Gnaq/11 and RTKs could represent a molecular driver of the growth of melanocytic cells in this specific anatomical location. Our findings contrast observations of a negative selection pressure from interfollicular epidermal keratinocytes against melanocytes harboring Gnaq^Q209L^ mutations [33]. We hypothesize that the reciprocal interaction of oncogenic driver mutations, genomic structural aberrations, and microenvironmental cues on key signaling pathways determines the anatomical compartments of tumor growth [34]. The microenvironmental modulation of oncogenic signaling cascades might also underlie the tissue-dependent responses to targeted therapy approaches in different cancer entities and pose limitations to therapeutic strategies focusing primarily on driver mutations, e.g., performed in clinical trials such as the NCI-MATCH project [35]. Our results, therefore, suggest that a more thorough understanding of these mechanisms is required to fully leverage the arsenal of therapies targeting signaling cascades in cancer.

### Supplementary information

Supplementary Figure 1. Selection of Gnaq/11 Q209 and Trp53 mutations during serial transplantation.

Supplementary Figure 2. Comprehensive analysis of signaling pathways after Gnaq inhibition.

Supplementary Figure 3. Cytotoxicity of uveal melanoma cell lines exposed to MET inhibition.

Supplementary Table 1

## Data Availability

The data that support the findings of this study are available.
