# Supplementary figures and images for: Transactivation of Met signaling by oncogenic Gnaq drives the evolution of melanoma in Hgf-Cdk4 mice
Source: Cancer Gene Ther. 2024 Feb 15;31(6):884–93. doi: 10.1038/s41417-024-00744-0 (PMC11192630; doi:10.1038/s41417-024-00744-0)

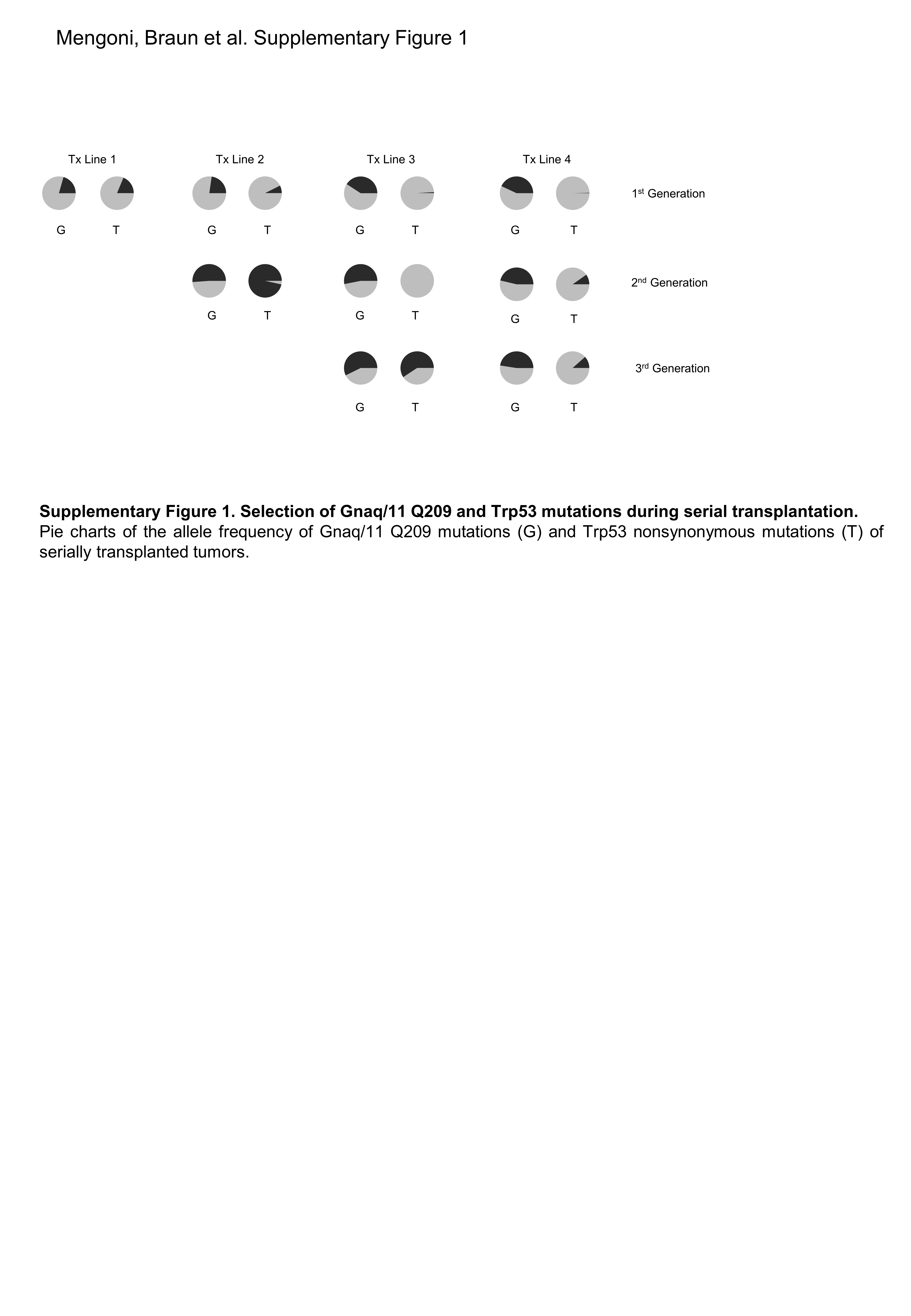

Supplement: Supplementary file 1 — Supplementary Figure 1. Selection of Gnaq/11 Q209 and Trp53 mutations during serial transplantation. [file 41417_2024_744_MOESM1_ESM.tif]

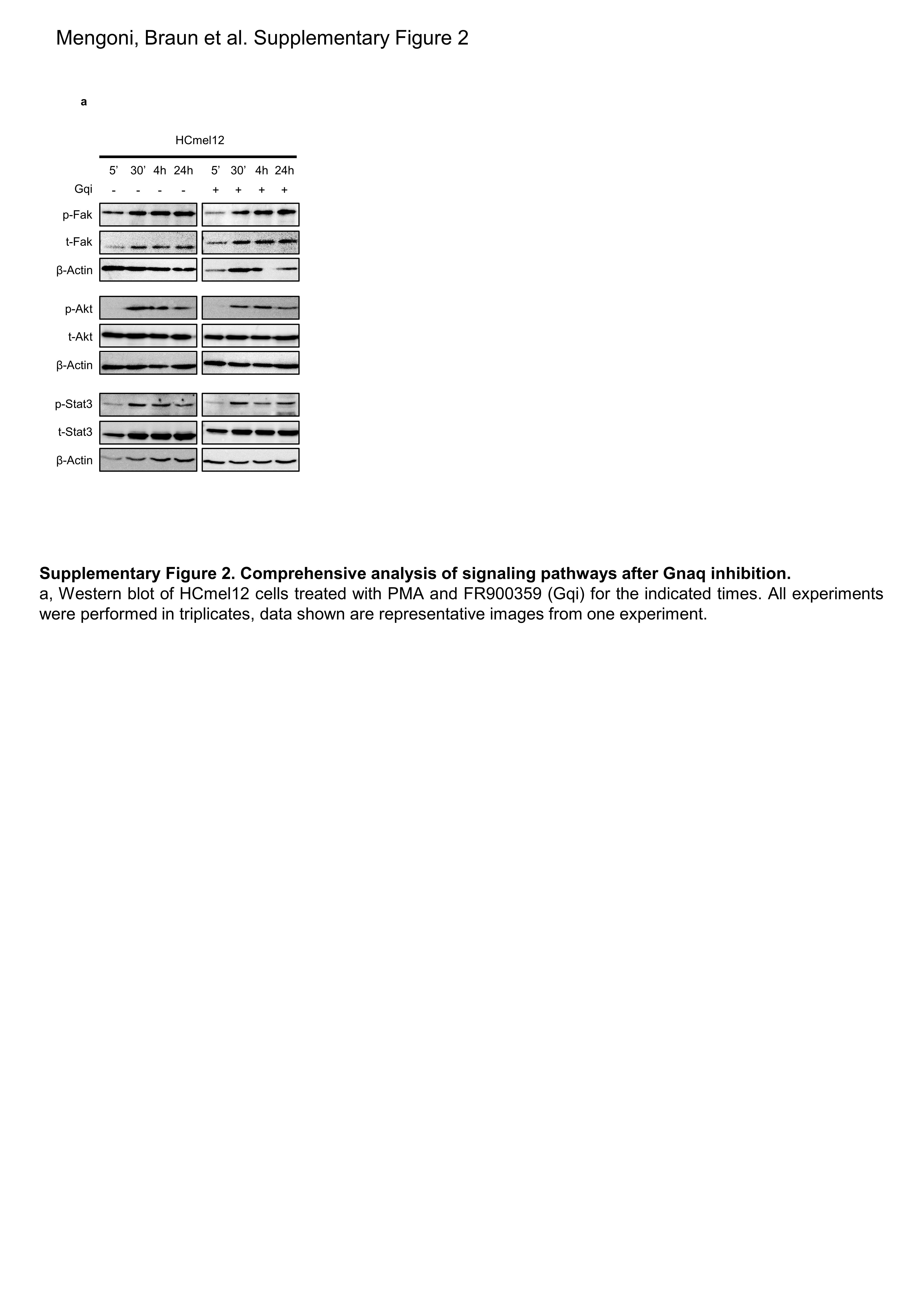

Supplement: Supplementary file 2 — Supplementary Figure 2. Comprehensive analysis of signaling pathways after Gnaq inhibition. [file 41417_2024_744_MOESM2_ESM.tif]

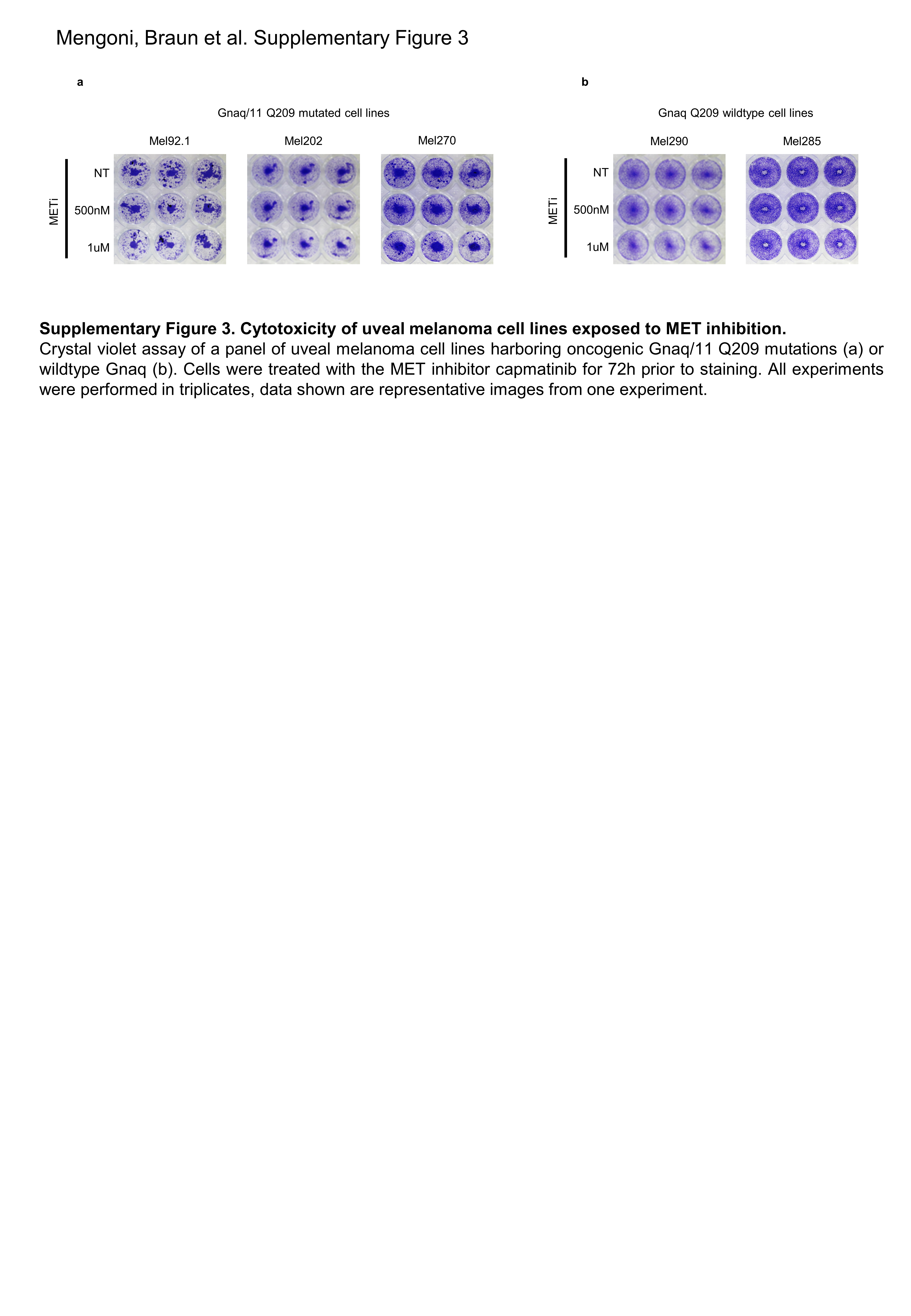

Supplement: Supplementary file 3 — Supplementary Figure 3. Cytotoxicity of uveal melanoma cell lines exposed to MET inhibition. [file 41417_2024_744_MOESM3_ESM.tif]
